# The Integrative Taxonomic Approach Reveals Host Specific Species in an Encyrtid Parasitoid Species Complex

**DOI:** 10.1371/journal.pone.0037655

**Published:** 2012-05-30

**Authors:** Douglas Chesters, Ying Wang, Fang Yu, Ming Bai, Tong-Xin Zhang, Hao-Yuan Hu, Chao-Dong Zhu, Cheng-De Li, Yan-Zhou Zhang

**Affiliations:** 1 Key Laboratory of Zoological Systematics and Evolution, Institute of Zoology, Chinese Academy of Sciences, Beijing, China; 2 Division of Forest Protection, School of Forestry, Northeast Forestry University, Harbin, China; 3 Ningbo Technology Extension Center for Forestry and Specialty Forest Products, Ningbo, China; 4 College of Life Science, Anhui Normal University, Wuhu, China; University of Guelph, Canada

## Abstract

Integrated taxonomy uses evidence from a number of different character types to delimit species and other natural groupings. While this approach has been advocated recently, and should be of particular utility in the case of diminutive insect parasitoids, there are relatively few examples of its application in these taxa. Here, we use an integrated framework to delimit independent lineages in *Encyrtus sasakii* (Hymenoptera: Chalcidoidea: Encyrtidae), a parasitoid morphospecies previously considered a host generalist. Sequence variation at the DNA barcode (cytochrome c oxidase I, COI) and nuclear 28S rDNA loci were compared to morphometric recordings and mating compatibility tests, among samples of this species complex collected from its four scale insect hosts, covering a broad geographic range of northern and central China. Our results reveal that *Encyrtus sasakii* comprises three lineages that, while sharing a similar morphology, are highly divergent at the molecular level. At the barcode locus, the median K2P molecular distance between individuals from three primary populations was found to be 11.3%, well outside the divergence usually observed between Chalcidoidea conspecifics (0.5%). Corroborative evidence that the genetic lineages represent independent species was found from mating tests, where compatibility was observed only within populations, and morphometric analysis, which found that despite apparent morphological homogeneity, populations clustered according to forewing shape. The independent lineages defined by the integrated analysis correspond to the three scale insect hosts, suggesting the presence of host specific cryptic species. The finding of hidden host specificity in this species complex demonstrates the critical role that DNA barcoding will increasingly play in revealing hidden biodiversity in taxa that present difficulties for traditional taxonomic approaches.

## Introduction

Parasitoids are insects that feed upon arthropod hosts during larval development [1]. They represent a key division of terrestrial food webs [2], [3], [4], and yet knowledge, particularly on their species richness, is severely limited [5], [6]. This situation is understandable given the lack of morphological differentiation in many sibling species, and the methodological difficulties posed in rearing due to the presence of multiple tropic levels, and complex life cycle [7], but must be addressed if factual estimates of insect diversity and host-specificity are to be known. Parasitoids represent a substantial proportion of biodiversity, with about 8.5% of described insect species [2], yet this figure does not take into account current thinking on the constraints of host parasite relationships [3], [8], [9], [10], meaning the diversity of parasitoids may be a substantial underestimation.

The discovery of cryptic species is proliferating in no small part due to the adoption of molecular data into taxonomic study. In particular, a new tool has been developed and is widely adopted and tested, that is providing invaluable information about species identities in such difficult to study taxa. DNA barcoding typically uses universal primers to sequence a standardized segment of the mitochondrial COI gene [11]. The resulting data can be used in i) assigning taxon names to newly sequenced individuals, by reference to a barcode library, and more controversially, ii) delimiting species boundaries and thus assigning new species. Considerable investment has been made to the barcoding endeavor, with the barcode of life database (BOLD) currently holding over 110,000 species, with the eventual aim to obtain 10× coverage for all ∼ 10 million animal species [12]. The ease and rate at which barcode sequences are being obtained and analyzed mean they have been of great utility in highlighting possible cases of cryptic speciation, often prompting further taxonomic work [13], [14], [15], [16], [17], [18], [19], [20]. In the case of cryptic parasitic species, it is often found that the sibling populations correspond to differing hosts species [21], [22], [23], suggesting that host generalism has been assumed where it is unwarranted. Theory suggests generalism (host generalism and otherwise) is unlikely to be maintained though speciation [10], meaning apparent examples of generalism are illusory, and thus current biodiversity estimates are an underestimation [24]. Given the breadth of inquiries and biological endeavors that may be sensitive to the accurate description of species, and the power of DNA barcoding to provide extensive divergence information with little expertise or taxon specific knowledge, it seems inevitable that taxonomic description will incorporate barcoding-like approaches, and that patterns in host-parasite relationships will be better resolved.

**Figure 1:**
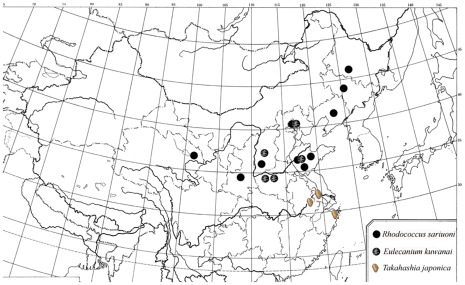
Host sampling sites.

While DNA barcode datasets sweep through biodiversity, few would advocate replacing current species descriptions with groupings defined by sequence variation from a single fragment of mitochondrial DNA. No particular approach to taxonomy is without complication, and the theoretic causes of incongruence between mitochondrial variation and a species tree are well known [25]. There is intuitive benefit in taking a whole evidence, or ‘integrative’ approach to taxonomy [26], and consult evidence from different disciplines in order to avoid pitfalls associated with a single approach. Incongruence between methods arises from various aspects. Firstly, while a general consensus is emerging on a definition of the species [27], disagreements remain on the degree of divergence at which separately evolving populations are regarded as different species [28], [29]. In addition, the evolutionary processes resulting in population divergence are heterogeneous [30]. The integrative taxonomy approach uses numerous such lines of evidence to corroborate taxonomic hypotheses, without ruling out that a single delineation criterion may correctly indicate the species [26]. Commonly used delimitation criteria include phenotypic distinctiveness, ecological niche divergence [31], reciprocal monophyly [32] and clustering of molecular data [33]. For example, extensive mitochondrial variation alone cannot be used to infer species, where reproductive compatibility is still present [34]. In the current paper we take an integrative approach to delineate species in the *E. sasakii* complex. *E. sasakii* are endoparasitic Hymenoptera belonging to the hyperdiverse wasp family, Encyrtidae (Hymenoptera: Chalcidcoidea). The hosts of *E. sasakii* are scale insects (of the Coccoidea superfamily), specifically, *Rhodococcus sariuoni, Takahashia japonica, Eulecanium kuwanai* and *Eulecanium gigiantea* [35], [36], [37], [38], [39], [40]. We find evidence of extensive molecular variation at the barcode locus among *E. sasakii* populations inhabiting different hosts, and find corroboration in the form of reproductive and morphometric characteristics.

## Methods

### Collection of Host Populations

In view of the broad range of hosts recorded for *E. sasakii* in the literature (see above), a survey of the hosts yielding *E. sasakii* was carried out during the period 2006–2010. However, only the host species *Eulecanium kuwanai* (Kuwana), *Eulecanium giganteum* (Shinji), *Takahashia japonica* (Cockerell) and *Rhodococcus sariuoni* generated the *E. sasakii* parasitoid. These host species are distributed in central and northern China, Japan (*T. japonica*) and Korea (*E. kuwanai*). In total, 18 populations of the host species were collected from host plants (*Sophora japonica, Lorpetalum chinense, Ulmus* sp. etc), throughout their continental range (Figure 1). Twigs from scale insect infested plants were returned to the lab and parasitoids segregated upon emergence. ∼2000 *E. sasakii* individuals were lab reared. Parasitoids were identified by author Yan-Zhou Zhang. The host scale insects were identified by an experienced taxonomist, Professor San-An Wu.

### Ethics Statement

No specific permits were required for the described field studies.

### DNA Extraction, PCR and Sequencing

DNA was extracted from adult specimens using the DNeasy Blood & Tissue Kit (Qiagen) according to the manufacturer’s protocols. All PCRs were performed on an Eppendorf thermal cycler, using 50 µL reaction volume as follows: 5 µL DNA template, 5 µL 10× Buffer (Takara), 25 mM MgCl2, 2.5 mM dNTP mixture, 10 pmol of each primer, and 1 unit of ExTaq DNA polymerase (Takara). To amplify 28S ribosomal gene D2 expansion segment, the primers D2-3549 [F] 5′- AGTCGTGTTGCTTGATAGTGCAG -3′ [41] and D2-4068[R] 5′-TTGGTCCGTGTTTCAAGACGGG-3′ [42] were used. PCR cycles were as follows: 3 min at 94°C; 30 cycles of 1 min at 94°C, 45 s at 58C, 1 min at 72°C; followed by 6 min at 72°C. The mitochondrial cytochrome oxidase I (COI) gene was amplified using the universal DNA barcoding primers LCO1490 (5′-GGTCAACAAATCATAAAGATATTGG-3′), and HCO2198 (5′-TAAACTTCAGGGTGACCA) [43]. The PCR program was as follows: 1 cycle of 3 min at 94°C, 5 cycles of 1 min at 94°C, 1 min at 45°C, and 1.5 min at 72°C, followed by 30 cycles of 1 min at 94°C, 1 min at 50°C, and 1 min at 72°C, with a final step of 5 min at 72°C. PCR products were electrophoresed through agarose gel (1%) then sequenced using BigDye v3.1 on an ABI PRISM 3730×l DNA Analyzer.

**Figure 2:**
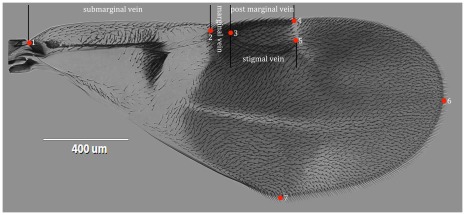
Forewing of *E. sasakii*, showing positions of the seven landmarks used for morphometric analyses.

### Analysis of Molecular Data

Sequence alignment was unambiguous, and carried out manually using BioEdit [44]. Model testing was performed on individual partitions, and the concatenated matrix, using MrAIC v1.4.3 [45] and PhyML v2.4.4 [46]. Phylogenies were then inferred under the optimal evolutionary model using MrBayes v3.1.2 [47]. Evolutionary parameters (state frequencies, substitution rates, alpha and the proportion of invariant sites) were allowed to vary amongst four partitions; 28 s, and the three codon positions of COI. Two independent runs were performed, both with one cold and seven heated chains, and sampled at intervals of 10,000. Runs were terminated when the standard deviation of split frequencies dropped below 0.01, then the parameter distributions checked using Tracer v1.5 [48]. Neighbor joining trees were also generated under the optimal model, using Paup*4b [49]. The branch-lengths on the Bayesian phylogeny and the NJ phylogram were adjusted by non-parametric rate smoothing [50] to form an ultrametric tree for analysis of branch waiting times. Branch rate smoothing was carried out using the r8s program [51], fixing the age of the root node at an arbitrary value of 1.0. The evolutionary units on the ultrametric trees were then inferred using the general mixed Yule coalescent approach (GMYC) [52], with a likelihood ratio test performed of a GMYC model against a null model whereby a single coalescent population was fit upon the tree.

**Figure 3:**
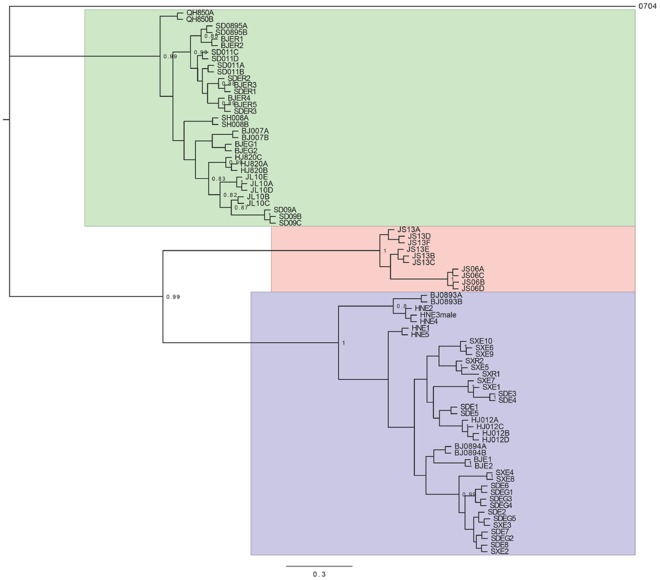
Bayesian consensus phylogeny of *E. sasakii*. Node support is indicated by posterior probabilities, and is given where >80. The upper (green), central (red), and lower (blue) clade represent specimens isolated from *R. sariuoni*, *T. japonica*, and *E. kuwanai/E. gigiantea*, respectively. First two letters of terminal name indicate sampling locality, where QH = Qinhai, SD = Shandong, BJ = Beijing, SH = Shaanxi, HJ = Heilongjiang, JL = Jilin, JS = Jiangsu, HN = Henan, SX = Shanxi.

**Figure 4:**
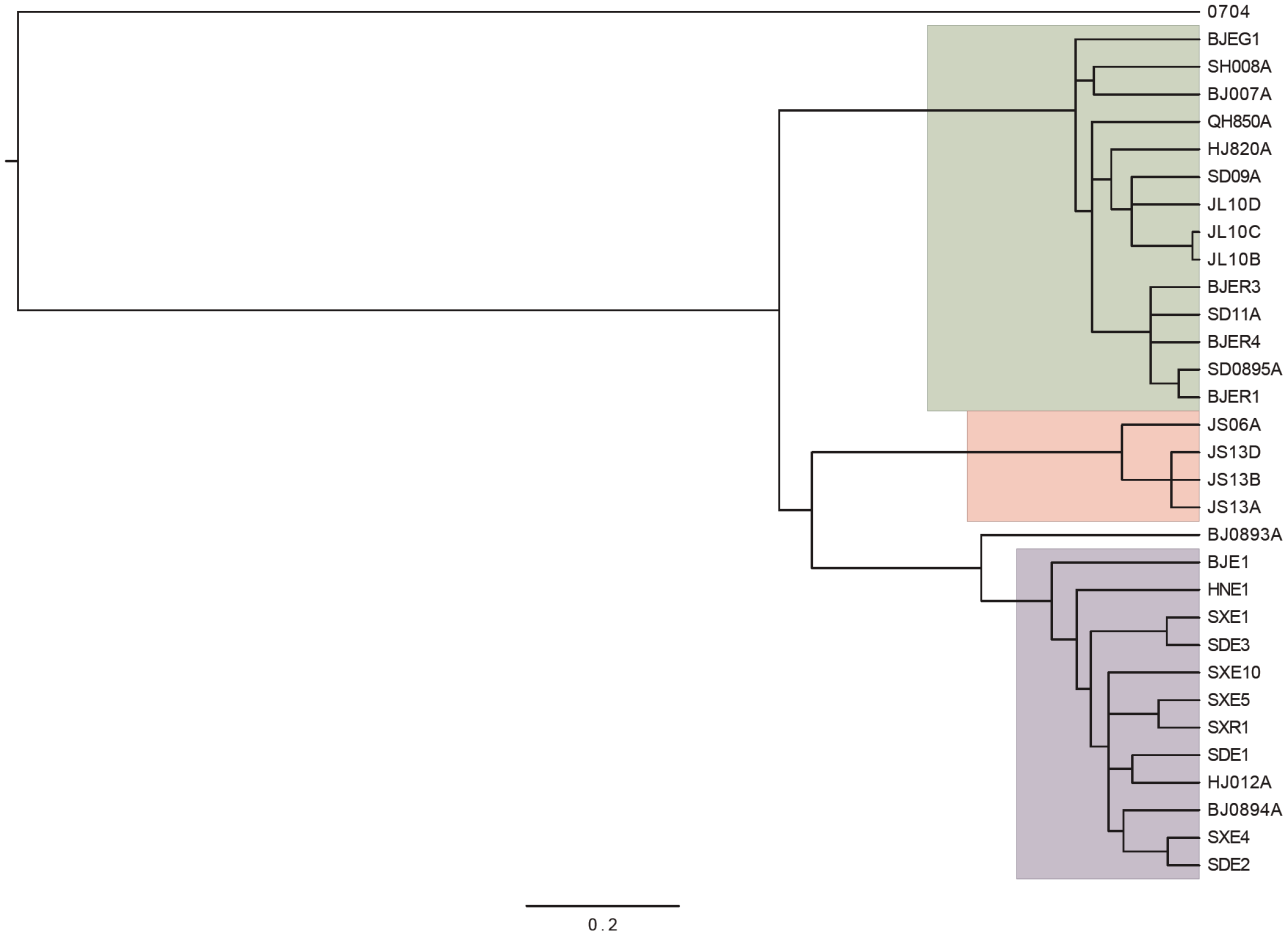
GMYC groups on the ultrametric NJ tree, generated from 31 unique haplotypes. Three clusters (shown in highlighted boxes) and one singleton (BJ0893A) are found as significant GMYC entities.

The molecular distances between individuals from different populations were calculated by the standard K2P measure for DNA barcodes, using Paup*4b, and characters diagnosing the populations identified using the Caos software [53]. The distribution of molecular divergences found between the populations was compared to divergences in Chalcidoidea as a whole, using i) intraspecific divergences, and ii) congeneric divergences. All Chalcidoidea DNA sequences were downloaded from Genbank, and searched locally using software from the Blast+ toolkit [54]. A Chalcidoidea database was created with makeblastdb, and queried using one of the newly sequenced *E. sasakii* COI sequences (JS06A). The blastn method was used for homology searching, with a strict e-value cutoff of 1e-5, and the tabular output format invoked (option: -outfmt 6) to aid parsing. The hit sequences were then extracted and a fasta file formed, using a Perl script. The COI barcode sequences were then aligned using the protein version of BlastAlign [55], against the translated JS06A sequence. The aligned Chalcidoidea sequences were checked by eye and the edges trimmed, using BioEdit. Where species were fully identified (where the species string in the description line matched the typical binomial format), the K2P distances were calculated as previously. The molecular distances were then split into intraspecific observations, and congeneric observations. The *E. sasakii* and Chalcidoidea distances were read into R for analysis [56].

**Table 1:** Specimens information on the sequences used in molecular analyses.

ID	Insect Host	Sampled Location	Plant Host
704	soft scale	Hainan, Danzhou	*Hibiscus rosa-sinensis*
BJE1	*E. kuwanai*	Beijing, Haidian	*Sophora japonica*
BJE2	*E. kuwanai*	Beijing, Haidian	*Sophora japonica*
BJ0893A	*E. kuwanai*	Beijing, Xiangshan	*Sophora japonica*
BJ0893B	*E. kuwanai*	Beijing, Xiangshan	*Sophora japonica*
BJ0894A	*E. kuwanai*	Beijing, Xiangshan	*Sophora japonica*
BJ0894B	*E. kuwanai*	Beijing, Xiangshan	*Sophora japonica*
HJ012A	*E. kuwanai*	Heilongjiang, Harbin	*Ulmus* sp.
HJ012B	*E. kuwanai*	Heilongjiang, Harbin	*Ulmus* sp.
HJ012C	*E. kuwanai*	Heilongjiang, Harbin	*Ulmus* sp.
HJ012D	*E. kuwanai*	Heilongjiang, Harbin	*Ulmus* sp.
HNE1	*E. kuwanai*	Henan: Zhengzhou	*Sophora japonica*
HNE2	*E. kuwanai*	Henan: Zhengzhou	*Sophora japonica*
HNE3	*E. kuwanai*	Henan: Zhengzhou	*Sophora japonica*
HNE4	*E. kuwanai*	Henan: Zhengzhou	*Sophora japonica*
HNE5	*E. kuwanai*	Henan: Zhengzhou	*Sophora japonica*
SDE1	*E. kuwanai*	Henan: Zhengzhou	*Sophora japonica*
SDE2	*E. kuwanai*	Shandong: Taian	*Sophora japonica*
SDE3	*E. kuwanai*	Shandong: Taian	*Sophora japonica*
SDE4	*E. kuwanai*	Shandong: Taian	*Sophora japonica*
SDE5	*E. kuwanai*	Shandong: Taian	*Sophora japonica*
SDE6	*E. kuwanai*	Shandong: Taian	*Sophora japonica*
SDE7	*E. kuwanai*	Shandong: Taian	*Sophora japonica*
SDE8	*E. kuwanai*	Shandong: Taian	*Sophora japonica*
SDEG1	*E. gigantean*	Shandong: Taian	*Albizzia julibrissn*
SDEG2	*E. gigantean*	Shandong: Taian	*Albizzia julibrissn*
SDEG3	*E. gigantean*	Shandong: Taian	*Albizzia julibrissn*
SDEG4	*E. gigantean*	Shandong: Taian	*Albizzia julibrissn*
SDEG5	*E. gigantean*	Shandong: Taian	*Albizzia julibrissn*
SXE1	*E. kuwanai*	Shanxi: Taiyuan	*Sophora japonica*
SXE2	*E. kuwanai*	Shanxi: Taiyuan	*Sophora japonica*
SXE3	*E. kuwanai*	Shanxi: Taiyuan	*Sophora japonica*
SXE4	*E. kuwanai*	Shanxi: Taiyuan	*Sophora japonica*
SXE5	*E. kuwanai*	Shanxi: Taiyuan	*Sophora japonica*
SXE6	*E. kuwanai*	Shanxi: Taiyuan	*Sophora japonica*
SXE7	*E. kuwanai*	Shanxi: Taiyuan	*Sophora japonica*
SXE8	*E. kuwanai*	Shanxi: Taiyuan	*Sophora japonica*
SXE9	*E. kuwanai*	Shanxi: Taiyuan	*Sophora japonica*
SXE10	*E. kuwanai*	Shanxi: Taiyuan	*Sophora japonica*
SXR1	*E. kuwanai*	Shanxi: Taiyuan	*Sophora japonica*
SXR2	*E. kuwanai*	Shanxi: Taiyuan	*Sophora japonica*
JS06A	*T. japonica*	Jiangsu, Nanjing	*Albizzia julibrissn*
JS06B	*T. japonica*	Jiangsu, Nanjing	*Albizzia julibrissn*
JS06C	*T. japonica*	Jiangsu, Nanjing	*Albizzia julibrissn*
JS06D	*T. japonica*	Jiangsu, Nanjing	*Albizzia julibrissn*
JS13A	*T. japonica*	Zhejiang, Ningbo	*Lorpetalum chinense*
JS13B	*T. japonica*	Zhejiang, Ningbo	*Lorpetalum chinense*
JS13C	*T. japonica*	Zhejiang, Ningbo	*Lorpetalum chinense*
JS13D	*T. japonica*	Zhejiang, Ningbo	*Lorpetalum chinense*
JS13E	*T. japonica*	Zhejiang, Ningbo	*Lorpetalum chinense*
JS13F	*T. japonica*	Zhejiang, Ningbo	*Lorpetalum chinense*
BJ007A	*R. sariuoni*	Beijing, Haidian	*Malus spectabilis*
BJ007B	*R. sariuoni*	Beijing, Haidian	*Malus spectabilis*
BJEG1	*R. sariuoni*	Beijing, Xiang Shan	*Ulmus* sp.
BJEG2	*R. sariuoni*	Beijing, Xiang Shan	*Ulmus* sp.
BJER1	*R. sariuoni*	Beijing, Haidian	*Malus spectabilis*
BJER2	*R. sariuoni*	Beijing, Haidian	*Malus spectabilis*
BJER3	*R. sariuoni*	Beijing, Haidian	*Malus spectabilis*
BJER4	*R. sariuoni*	Beijing, Haidian	*Malus spectabilis*
BJER5	*R. sariuoni*	Beijing, Haidian	*Malus spectabilis*
HJ820A	*R. sariuoni*	Heilongjiang, Harbin	*Prunus persica*
HJ820B	*R. sariuoni*	Heilongjiang, Harbin	*Prunus persica*
HJ820C	*R. sariuoni*	Heilongjiang, Harbin	*Prunus persica*
JL10A	*R. sariuoni*	Jilin, Changchun	*Prunus persica*
JL10B	*R. sariuoni*	Jilin, Changchun	*Prunus persica*
JL10C	*R. sariuoni*	Jilin, Changchun	*Prunus persica*
JL10D	*R. sariuoni*	Jilin, Changchun	*Prunus persica*
JL10E	*R. sariuoni*	Jilin, Changchun	*Prunus persica*
QH850A	*R. sariuoni*	Qinghai, Xining	*Prunus persica*
QH850B	*R. sariuoni*	Qinghai, Xining	*Prunus persica*
SD0895A	*R. sariuoni*	Shandong, Tai’an	*Prunus cerasifera*
SD0895B	*R. sariuoni*	Shandong, Tai’an	*Prunus cerasifera*
SD09A	*R. sariuoni*	Shandong, Linyi	*Crataegus pinnatifida*
SD09B	*R. sariuoni*	Shandong, Linyi	*Crataegus pinnatifida*
SD09C	*R. sariuoni*	Shandong, Linyi	*Crataegus pinnatifida*
SD011A	*R. sariuoni*	Shandong, Linyi	*Prunus persica*
SD011B	*R. sariuoni*	Shandong, Linyi	*Prunus persica*
SD011C	*R. sariuoni*	Shandong, Linyi	*Prunus persica*
SD011D	*R. sariuoni*	Shandong, Linyi	*Prunus persica*
SDER1	*R. sariuoni*	Shandong, Tai’an	*Prunus cerasifera*
SDER2	*R. sariuoni*	Shandong, Tai’an	*Prunus cerasifera*
SDER3	*R. sariuoni*	Shandong, Tai’an	*Prunus cerasifera*
SH008A	*R. sariuoni*	Shaanxi, Xianyang	*Malus* sp.
SH008B	*R. sariuoni*	Shaanxi, Xianyang	*Malus* sp.

**Figure 5:**
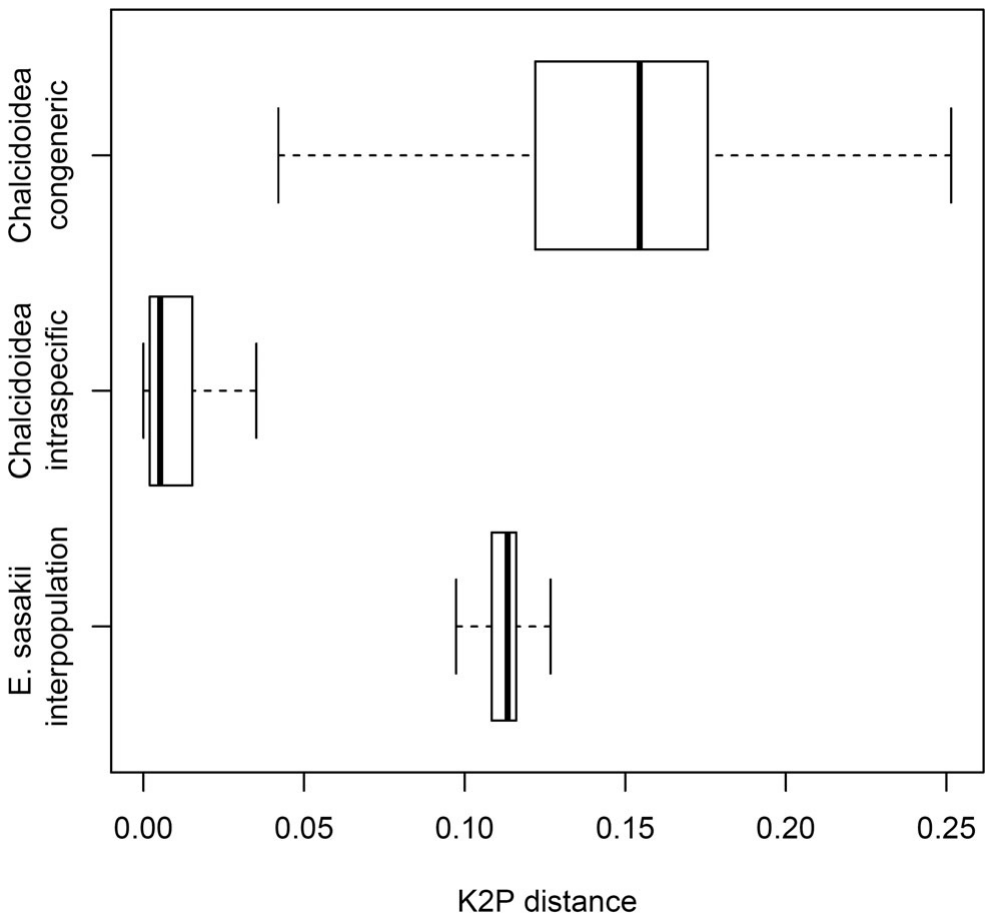
Boxplot giving pair-wise molecular distances between, (upper) individuals from different species of the same genus in the Chalcidoidea, (central) different members of the same species in the Chalcidoidea, (lower) individuals belonging to different *E. sasakii* host-related populations.

### Morphometric Analysis

Geometric morphometrics have been used to study various insect taxa ranging from species level to analysis of a superfamily, and have been informative in investigating relationships between members of lower taxonomic levels [57]. In this study, the first application of geometric morphometrics in Encyrtidae was carried out. Although previous taxonomy of the genus *Encyrtus* [58], [59] has focused on the shape of both the antenna and its forewing, due to high variation and the difficulty in preparing of slide mounted antennae, here only the forewings are used. In total, 59 specimens were prepared for geometric morphometric analysis, using individuals randomly selected from those used for DNA extraction, and covering all populations. The specimens were dissected and examined using a Leica MZ12.5 stereoscope. The microphotographs were taken from slide mounted specimens using an EVOS f1 inverted microscope. Seven landmarks were selected to describe variation in wing morphology (Figure 2). The landmarks were as follows: 1, the beginning of submarginal vein; 2, the end of submarginal vein/beginning of marginal vein; 3, the end of marginal vein/beginning of post marginal vein/beginning of stigmal vein; 4, the end of postmarginal vein; 5, the end of stigmal vein; 6, the tip of forewing; 7, the tip of posterior margin of forewing. Cartesian coordinates of the landmarks were digitized with tps-DIG 2.05 [60]. In order to reduce the measurement error all specimens were digitized twice. The coordinates were analyzed using tps-RELW 1.44 [61] to calculate eigen values for each principal warp. Statistical analyses were performed using SPSS version 16.0 for windows [62].

### Mating Tests

The courtship and mating behaviors of *E. sasakii* intrapopulation and interpopulation pairs were observed through reciprocal crosses. Crosses were performed during the period of host emergence overlap (May). Virgin individuals were paired in vials (one male and one female per vial) and observed for 7 days, with 10 replicates performed for each of the nine possible reciprocal population combinations. A solution of bee honey (50%) was provided as food supply during the mating tests.

## Results

### Analysis of Molecular Data

Fragments for COI and 28S were successfully sequenced for 83 *E. sasakii* specimens, from 18 populations plus the outgroup *Encyrtus auranti* shown as 0704, in Figures 3 and 4 (detailed information see Table 1). After edge trimming, the data matrix consisted of 631 base pairs for COI and 511 bases for 28S. The 28S gene was virtually invariant for the sequenced specimens, however it contained a single base substitution (at site 205), with the cytosine character unique to samples obtained from the host *R. sariuoni*, and thymine for samples obtained from hosts *T. japonica* and *E. kuwanai*. Typically for insect mitochondrial genes, the AT content was high (68.8%), however, at the lower end of the range compared to other parasitic wasps, e.g. 74.85% in Cynipidae [63], 74.0% in Apocrita [64], 72% in Eulophidae [65], 68% in Braconidae [66].

The degree of genetic divergence in COI was found to be particularly high between the three populations. The mean K2P distance between pairs belong to different *E. sasakii* populations was 11.24%, with 1.5% divergence within populations. In order to determine if this was significantly high compared with species in the superfamily as a whole, 2393 Chalcidoidea barcode sequences (225 fully identified species and 77 genera) were downloaded from Genbank and aligned, then K2P distances for two classes (intraspecific and congeneric) were calculated. Figure 5 plots K2P values for the Chalcidoidea, along with the divergences between the three *E. sasakii* populations. While the *E. sasakii* molecular divergences do not belong to either the intraspecific or congeneric Chalcidoidea distributions (p<0.001 in both cases, unpaired Wilcoxon signed rank test), the median *E. sasakii* divergence (0.113) is over an order of magnitude higher than the median Chalcidoidea intraspecific divergence (0.005), and well within the same order of magnitude than the median Chalcidoidea congeneric divergence (0.155), indicating the *E. sasakii* populations show molecular variation more representative of congeners.

**Figure 6:**
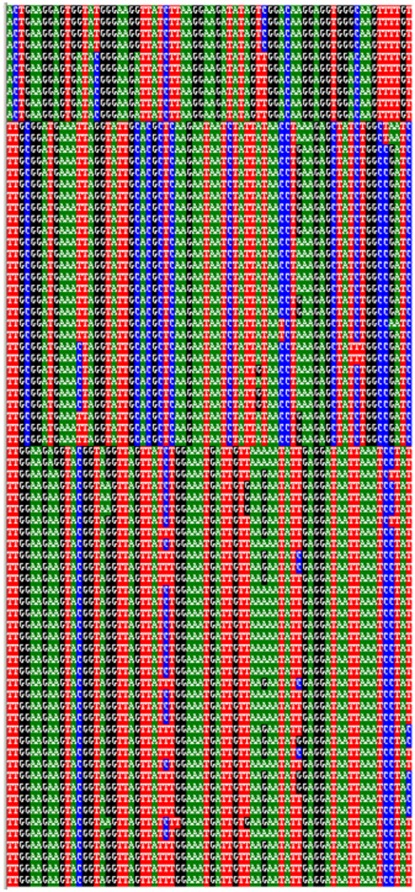
The 73 pure diagnostic characters isolated from the COI alignment.

Characters diagnostic of the three main populations were identified using Caos. 122 (19.4% of the COI positions) were found diagnosing one or more of the populations, where all the characters were classed as simple (non-compound). These 122 sites were subdivided into 73 pure (unique to all members of the clade) and 49 private (present in some clade members but absent in other clade) positions. Figure 6 gives a graphic illustration of the 73 pure diagnostic characters when isolated from the dataset, and a table giving the total 298 characters (with population identity, diagnostic character state, position and confidence value) is provided in the supplementary file (File S1).

**Figure 7:**
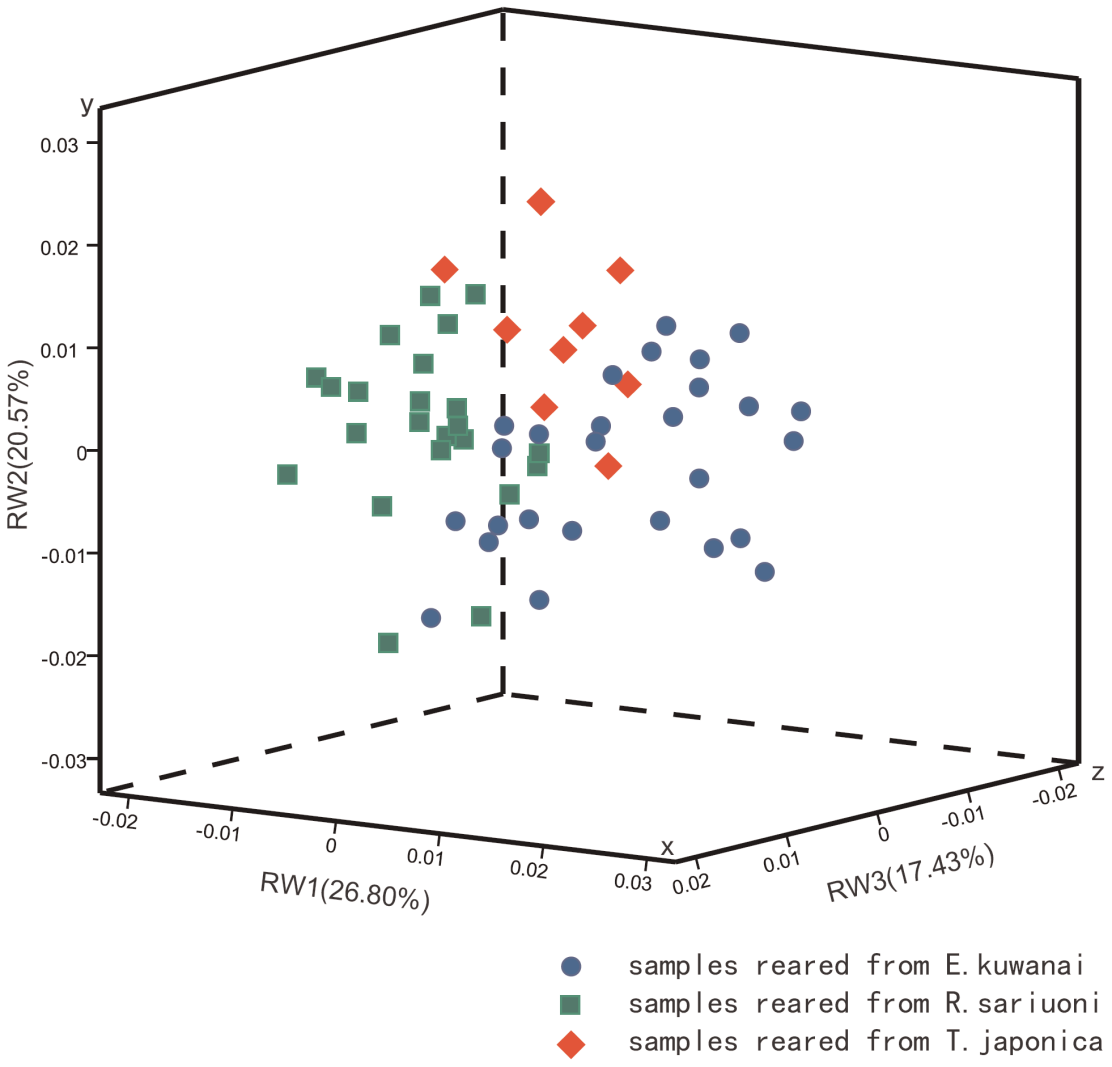
Three-D scatter plots constructed from principal component analyses of the landmark data set. In the scatter plots the first, second and third principal components were plotted on the x (RW1), y (RW2) and z (RW3) axis respectively.

**Table 2:** Mating tests.

Female	Male		
	*RS*	*EK*	*TJ*
*RS*	+	–	–
*EK*	–	+	–
*TJ*	–	–	+

*RS*, population of *Encyrtus sasakii* reared from *R. sariuoni*, *EK*, population of *Encyrtus sasakii* reared from *E. kuwanai*, *TJ*, population of *Encyrtus sasakii* reared from *T. japonica*; “+” indicates the observation of courtship and mating behavior in at least one replicate, “–” indicates no courtship or mating behavior observed throughout the testing period. Diagonals give intra-population crosses, with inter-population crosses otherwise.

The molecular data were subject to evolutionary analyses using NJ and Bayesian approaches. Due to the low number of parameters and low variation in some partitions (28S in particular), we used the AICc to determine the best fit model for the un-partitioned dataset, which was found to be the general time reversible with gamma distributed rates (d.f. 174, lnL -3156, AICc 6723, wAICc 0.71). Two independent MrBayes runs successfully converged (the standard deviation of split frequencies <0.01) after 12,950,000 generations. The parameters were checked in Tracer, where the estimated sample sizes were >200 in virtually all cases. The tree was summarized after discarding the burnin phase (25%), and shown in Figure 3. Three monophyletic clades were recovered corresponding to the three host populations, each with high posterior probabilities, and long subtending branch lengths. The host specificity was found to be complete in that all specimens within a clade were reared from the same host, without exception.

We next determined whether the pattern of branch lengths in the trees were characteristic of both within and between species branching events. For the Bayesian tree, we found no significant shift between Yule and Coalescent branch waiting times (lnL of GMYC model  = 393.5, lnL of null model  = 392.8, likelihood ratio  = 1.44, p = 0.70). We also performed this analysis on a NJ tree; unique haplotypes were isolated from the dataset, and a NJ tree generated under the GTR gamma model. As shown in Figure 4, the three significant GMYC clusters corresponded to the host associated groups apart from one sequence (BJ0893A) excluded from the *E. kuwanai* associated cluster (the lower blue colored clade in Figure 4). The GMYC model was a significant improvement in fit, over the null model of a single coalescent cluster (null lnL = 104, GMYC lnL = 110, likelihood ration = 12, p = 0.007), indicating the shift to longer branches separating the *E. sasakii* populations are characteristic of a change to interspecies branch waiting times.

### Morphometric Analysis

The relative warps analysis and cluster analysis of forewing shape revealed a trend dividing the populations into three host associated groups (Figure 7). The contribution of the 1st, 2nd, 3rd and 4th canonical variates to the total variance was 26.8, 20.57, 17.4 and 13.02 percent, respectively. To ensure reliability of the results, the first ten canonical variates were used for cluster analysis in SPSS 16.0. Analysis of Variance (ANOVA) tests were performed to determine population differences in forewing shapes. The three host clusters were significantly distinct in the first (p<0.01; F = 43.117; d.f. = 2), second (P<0.05; F = 3.527; d.f. = 2), and third variates (p<0.01; F = 13.56, d.f. = 2).

### Mating Test

Courtship and mating behavior were recorded as they occurred, in reciprocal crosses for all combinations of the three *E. sasakii* populations. Typical receptive behavior consisted of antennal contact followed by copulation [67], and repellence fighting occurred when the female was unreceptive. Courtship and mating behavior were observed in intra-population crosses only, never in inter-population crosses (Table 2), indicating pre-copulatory barriers to gene flow between host-specific populations.

## Discussion

### Barcode Divergence, Molecular Delineation and Identification

The likely case of cryptic speciation in *E. sasakii* was initially made apparent during routine DNA barcode sequencing, and the molecular evidence supporting the promotion of the host-specific populations to species level remains particularly striking. The degree of molecular divergences at the COI barcode locus, fell well outside the expected distribution for individuals of the same species (Figure 5). The inter-population divergence (11.24%) was found to be an order of magnitude higher than within-population divergence (1.5%), consistent with the barcode species criterion given by Hebert *et al.* [13]. But the major advantage of quantifying absolute level of divergence for COI in particular is the comprehensive benchmarks available in the literature. Hebert *et al.* [11], reported K2P divergence for Lepidoptera families as 0.17–0.33% for within species and 5.8–9.1% within genera. Ball *et al.* [68] gave 1.1% for within species and 18.1% for congeners in mayflies. Molbo *et al.* [69] discovered cryptic species where molecular divergence was 4.2–6.6% (amongst other lines of evidence). In a comprehensive analysis of barcode divergence using a number of mined insect datasets, Meier *et al.* [70] reported mean intraspecific/interspecific divergences as 2/11.2 for Coleoptera, 1.3/10.1 in the Diptera, 1.8/9.3 for Hymenoptera, and 0.7/6.2 for Lepidoptera, amongst others. While the intraspecific and congeneric divergences appear somewhat limited in their ability to vary across taxonomic groups, we thought it prudent to calculate specific values for the inclusive clade in which sufficient data were available. Figure 5 shows that the divergences between populations of *E. sasakii* are more representative of congeneric Chalcidoidea than intraspecific.

Given the molecular divergences, and the other advantages of barcode identification (e.g. ease of sequencing and non-requirement of taxon specific expertise), we suggest it warrants the adoption of molecular identification in this species complex. It has been demonstrated here that the properties of COI make it amenable to a number of proposed barcoding methods. The structuring of genetic variation makes the COI barcode an ideal marker for identification in this species complex, both due to the amount of divergence (Figure 5), and the robust reciprocal monophyly of the populations (Figure 3). The diagnostic characters given in the File S1 provide the rules for assignment of future query sequences to the newly proposed species. When used with algorithms such as Caos [53], such identification can be rapid and automatable.

Further analysis of the combined molecular data revealed that the three populations were recovered as robust monophyletic groups. Reciprocal monophyly requires fixation of divergent characters, these being typical of the later stages of lineage evolution [29]. However, further analysis of the shape of branching patterns was less clear-cut. The GMYC model tests for the presence of a shift from Yule (between species) to coalescent (within species) branch-lengths in an ultrametric tree, but was found significant for the NJ tree only. However, the choice of tree building method is likely a confounding factor for this test. Monaghan *et al.* [71] has previously noted the circularity of testing for a shift in branching pattern, on a tree that has been inferred under one of the very models being tested for. The imposition of root to tip branch length pattern during a tree search is very apparent using for example, the Beast software [48], where the default setting for branch-length model is coalescent, with additional options of Yule and birth-death. Preliminary analyses (not shown) were performed using this software, but these models has a clear bias on the resulting tree-shapes. A preferable approach would be tree inference independent of such models. In the current paper we applied the GMYC to a tree inferred under a Bayesian model in which branch lengths were unconstrained (non-clock), which precludes the imposition of root to tip branching model (which in the MrBayes clock trees include uniform, birth-death and coalescent), although the branch-lengths are sampled from a specified distribution (uniform or exponential). In an attempt to avoid all possible imposition of branch length bias we repeated the GMYC using a simple NJ tree, which was found to give significant GMYC groups. The analysis highlighted that where the aim is to analyze shift in these different types of branching it may be advisable to consult simpler tree building approaches, which may avoid some confounding effects.

### Integrative Taxonomy

Where the sample is limited (for example considering key species complexes), variance in the pattern of intra/inter species divergence appears greater [72], meaning the host correlated divergence observed in *E. sasakii* may simply represent a local increase in intraspecific variation. Confirmation that high divergence is the result of independent evolution should be obtained by reference to other character types. If other characters do not covary with molecular divergence, then it is not necessarily the case that multiple species are present [73]. The integrative approach to taxonomy overcomes biases associated individual lines of evidence and increases the information on which taxonomic hypotheses are tested [74]. Where corroborative evidence has been found from independent sources that support an alternative hypothesis, ‘breaking out’ of the current taxonomy is deemed reasonable [75]. Independent evidence may come from a number of sources, including various forms of molecular data, morphology, ecology, behavior, geography, and reproductive capacity [69], [74], [75], [76], [77]. Here, in addition to the molecular evidence, we show that i) the molecular clusters correspond to three clusters formed from certain morphometric characteristics, ii) these three putative taxonomic units inhabit differing niches (hosts), and iii) individuals from different hosts, when paired, show no mating capacity.

The hypothesis of cryptic species was further tested using morphometrics of the forewing. Forewing shape has been proposed as a morphometric-based population/species diagnostic character in the Hymenoptera, due to ease of slide preparation and high discriminatory power [78], [79], [80], [81], [82], [83]. In the current study we find the phenetic clusters based on the forewing shape are generally consistent with the phylogenetic classification, with both methods indicating differentiation according to host species. The populations isolated from the three hosts showed partially overlapping variation in wing pattern, reflecting the difficulties commonly encountered when analyzing morphological characters in sibling species groups [8]. However, the molecular divergence (Figure 5) and lack of courting or mating behavior between the *R. sariuoni* and *T. japonica* populations (Table 2) indicate these entities would be regarded as different species, according to many definitions of the concept. The unified species concept requires any method of delineation conforming to a single species concept in order to infer a species boundary, but where a delineation is congruent under multiple concepts (here for example, certainly the phylogenetic species concept and the biological species concept apply), the hypothesis can only be considered more robust [27].

### Cryptic Species and Host Specificity

There is an increasing number of cases where the initial analysis of molecular data has led to the discovery of previously unknown divergent features, but where parasitic taxa are under study, divergent populations usually corresponds to host specific races [84]. In *E. sasakii*, the three divergent genetic clusters (Figure 3) correspond to scale insect hosts, with geographic separation unlikely to have a substantial contribution to the molecular differentiation, since within clades, geographic sampling is widely ranged. For example the basal *R. sariuoni* associated clade (upper, green clade in Figure 3) contains samples obtained from regions ranging from central to far eastern China, covering areas sympatric with that of *E. kuwanai* associated parasitoids. This indicates the recent distribution of *E. sasakii* across much of the sampled range, whereas gene flow is prevented across different host groups, with all members of the basal clade isolated from a single scale insect species. While general conclusions can not be drawn based on this single species complex, there is a growing body of research indicating such host specificity is much more prevalent than previous diversity estimates suggest [10], [15], [18], [85], [86], [87], [88], [89], [90]. However, the route towards accurate estimates of diversity will be hindered by naive application of molecular sampling. As observed in *E. sasakii*, the presence of sympatric host races means informed approaches (particularly, using host identities) to the barcode sampling strategy are required to capture the diversity.

## Supporting Information

File S1 **COI diagnosing character states for** ***E. sasakii*** **populations.** Column ‘group’ gives population, ‘pos’ is COI site position, ‘state’ is diagnostic character state, and ‘conf’, confidence value.
(TXT)
